# A critical evaluation of science outreach via social media: its role and impact on scientists

**DOI:** 10.12688/f1000research.5918.2

**Published:** 2015-05-18

**Authors:** Craig McClain, Liz Neeley

**Affiliations:** 1 National Evolutionary Synthesis Center, Duke University, 2024 W. Main St. Durham, NC, 27712, USA; 2 COMPASS, University of Washington, College of the Environment, Box 355020 Seattle, WA, 98195, USA

**Keywords:** Social Media, Public Engagement, Science Outreach

## Abstract

The role of scientists in social media and its impact on their careers are not fully explored.  While policies and best practices are still fluid, it is concerning that discourse is often based on little to no data, and some arguments directly contradict the available data.  Here, we consider the relevant but subjective questions about science outreach via social media (SOSM), specifically: (1) Does a public relations nightmare exist for science?; (2) Why (or why aren’t) scientists engaging in social media?; (3) Are scientists using social media well?; and (4) Will social media benefit a scientist’s career? We call for the scientific community to create tangible plans that value, measure, and help manage scientists’ social media engagement.

## Introduction

Recently, both scientists and science communicators have issued numerous calls to the scientific community to engage in social media to both connect with other scientists (inreach) and to connect with the public (outreach). Many of the arguments made in support of science outreach via social media (SOSM) are simply special applications of arguments for outreach in general. However, researchers often unknowingly approach engagement and outreach with a variety of counterproductive misunderstandings and assumptions, including those about whether and why their peers do or do not engage. While we are limited by the scarcity of research on this topic, we are concerned that this leads to inaccurate conceptions of the value of SOSM based in assumptions and anecdotes as opposed to data. Here, we draw upon a variety of disciplines to address what is known of the role and impact of social media on scientists.

## A “public relations nightmare”?

Numerous calls for the scientific community to engage in science outreach via social media (SOSM) have been issued ^1, 2^ as part of a broader agenda for researchers to engage the public ^3– 5^. In particular, SOSM is presented as a promising new tool for science outreach in the midst of a perceived public relations crisis. Specific discussions of the public’s trust ^6^ and perceived legitimacy of scientists ^7^ have been enfolded into a “public relations nightmare” narrative ^1, 8, 9^. However, does science actually face diminished support or is the “public relations nightmare” an exaggeration reflecting a “institutional neurosis” of science practitioners? ^10^

Given the prominence of vocal anti-science movements in debates around high profile issues such as vaccines ^11, 12^, climate change ^13^, and evolution ^14, 15^—and especially considering the intensity and frequency of personal attacks on scientists in these arenas ^16, 17^—it is reasonable to ask whether the public fundamentally distrusts scientists. Despite the coverage of these anti-science movements, polls consistently find that scientists out-perform clergy, artists, journalists, business executives, and lawyers in perceived “contribution to society” ^18^. Even in the wake of the ClimateGate controversy, climate scientists remained more trustworthy than competing sources like weather reporters, political and religious leaders, or the mainstream media ^19^. Scientists are in demand for media interviews, political testimony, and public address, and their influence in policy arenas “is well documented, and so is public support for this arrangement” ^20^. In short, despite the evidence of growing political polarization ^21^, the cultural authority of organized science seems to have remained stable.

So why is the “public relations nightmare” narrative uncritically and continuously repeated? Besley and Nisbet ^22^ argue that scientists misunderstand public attitudes—despite the available data—because they regard non-scientists as hostile “others” who are ill-informed and highly susceptible to media messages, which they also perceive to be slanted against science. Some evidence does exist that these individual movements received disproportionate media coverage given their participatory numbers and impact/importance; and, in regard to both climate change denial and anti-vaccine movements, that “balanced” coverage sustains the claims in the public and disproportionately favors ideas running against scientific consensus ^23, 24^. However, the tendency to lump all opposition to science, despite topic, into a common “anti-science” camp with a single root cause, rather than recognizing the diversity of motivations for challenges and rejections creates further problems ^14, 25^. This them-versus-us narrative further solidifies a group entrenchment and contempt for outsiders which ultimately develops into a deeply emotional response. Interestingly, this group mentality does not match individual perceptions of the media; scientists rate personal interactions with media highly ^22^. With social media most scientists surveyed felt that the online public would likely benefit their career and the online audience would listen and treat them respectfully ^26^.

## Are scientists engaging in social media?

It is common to describe the scientific establishment as resistant to public engagement, and scientists tend not to believe that their colleagues actually engage in outreach ^27^. Yet surveys suggest that scientists engage in public communication activities more often than is commonly assumed ^28^. One survey of scientists found that nearly half of all academic scientists were engaged in some type of outreach ^29^ and interactions with mainstream media have become a standard expectation ^30, 31^.

Data and analyses on social media outreach are less forthcoming, but studies appear to indicate that uptake of social media by scientists is low. In a University of Michigan study, approximately 60% of the responding scientists engaged the media, public, and government agencies through traditional channels. Yet, nearly 40% of respondents stated they would never use Twitter for academic or professional work ^32^. Wilkinson and Wietkamp ^33^ surveyed researchers who highlighted their work in policy-relevant newsletters, reporting that, “For the majority of researchers, there has been little change in the use of [social] media to communicate with non-academic audiences over the past five years”. Of the respondents, 73% had never used Twitter, 64% had never used blogs, and 51% never engaged online news forums. These patterns of adoption offer a strange mismatch with the expressed beliefs of nearly 44% of German and 65% of American scientists who thought that social media channels, “can strongly influence how the public thinks about science” ^34^. It may be less a question of the platforms than intent. A 2010 survey of biology professionals and students, found that 73% of respondents did actively engage in social networking (broadly defined) as a research tool ^35^. A recent study ^36^, also confirms the relatively low use of Twitter, Facebook, and Google+ compared to the active use of Google Scholar (~60%) and ResearchGate (~40%).

Until recently, we have known little about the demographics of scientists engaging in public outreach through social media. In a 2012 survey of AAAS members ^35^, younger respondents were willing to participate in online engagement while women “were less likely to say they would be willing to write online articles, blog posts, or online responses”. Previous studies suggest that career, age, and gender differences lead to differences in outreach commitment. Twice as many female (83%) than male (43%) graduate students were involved in outreach ^37^. Ecklund *et al.* ^29^ found that women are markedly more involved in outreach work than men (72% versus 43%) and marked drop offs in public engagement occurs from graduate students to postdoctoral fellows and faculty. In contrast, male and female faculty members were more equally represented in science outreach based on their representation in the disciplines ^37^. Another study found that higher status and organization autonomy was also associated with greater outreach but found no effects with gender ^38^.

Overall, the best predictor of outreach by scientists appears to be linked to their perceptions of outreach. In general, scientists have a positive attitude toward participating in public engagement and fear associated with participation is low ^29^. There is also “high overall satisfaction of researchers with their own media contacts” ^30, 31^. Furthermore, scientists with more positive attitudes, higher perceived communication skills, and more formal communication training, are more likely to engage in these activities ^38^. Increased positive views toward public outreach also increase with increasing participation in these activities ^39^.

Perhaps the biggest obstacles to engagement with outreach in general and social media outreach specifically is a lack of time. Andrews and Weaver ^37^ found that time constraints were the unanimous primary impediment to scientists’ involvement in outreach. Participants’ availability to do outreach was limited by their other priorities, which were given precedence. Ecklund *et al.* ^29^ noted that for more than half of all scientists, a lack of time is the most insurmountable barrier to doing more outreach and perceived time constraints are associated with a more negative impression of doing outreach activities. For engagement with social media, Rowlands *et al.* ^35^ found that the most cited inhibitor was a lack of time.

## Are scientists communicating through social media well?

The view of scientists as poor communicators has been reinforced by media portrayals of scientists as eccentric and antisocial, possibly dangerous, and ultimately separate from the public ^40^. While negative stereotypes of scientists among American adults have decreased since the 1980s, about one quarter still agree that scientists are, “odd and peculiar” ^41^. Researchers are aware of these attitudes. Nearly 30% of scientists in one study agree that scientists are poor interpersonal communicators and more (37%) placed the blame on scientists themselves ^29^. However and importantly, no quantitative data or analyses support the perception that scientists are poor public communicators. We caution against the reliance on stereotypes. Arguing over anecdotal examples of scientists as both effective and ineffective communicators does little to identify legitimate deficiencies and offer effective remedies. What the scientific community desperately needs is objective studies on communication effectiveness of scientists and a clear definition of “poor communicator”. One tool of this nature is that of Baram-Tsabari and Lewenstein ^42^ that aims to assess scientists’ written skills in public communication. The framework includes assessment of clarity, content, knowledge organization, style, analogy, narrative, and dialogue.

## Will social media outreach benefit a scientist’s career?

With regard to social media outreach, the biggest reason given in informal discussions for a scientist’s involvement in social media outreach is that engagement will directly benefit a scientist’s career. We feel it important to reiterate that social media is a tool that can be utilized for purposes other than public engagement. Discussion of the benefits of social media inreach (engagement within the scientific community) and outreach (engagement with the public) are muddled.

Social media may be an important tool in quickly connecting with other researchers ^36, 43^. “This is the dilemma faced by researchers in the digital age. How can we be expected to produce both quality and quantity and to yield influential research? We simply cannot—at least not on our own. Instead, we must rely on networking and collaborations to build our research programs and to remain influential in our fields in order to advance scientific knowledge. With this collaborative view in mind, scientific influence involves the body of work of both individual researchers and of research groups as a whole” ^43^. Darling *et al.* ^44^ demonstrated that Twitter followers for scientists were always considerably larger than their departments and these large virtual departments help to rapidly generate, share and refine ideas. Moreover blogs written by scientists for scientists are becoming more common and serve as places for the exchange of ideas ^45^. However, the ratio of social media, such as blogs, by scientists for inreach verses outreach remains unclear.

In terms of social media outreach, or outreach in general, the impact on a scientist’s career remains largely unquantified and quite possibly indirect. “Many faculty members identified their primary job responsibilities as research and post-secondary teaching. They felt that outreach participation hindered their ability to fulfill those responsibilities and might be an ineffective use of their skills and time, and that it was not a valid use of their research funding ^37^”. In the survey by Ecklund *et al.* ^29^, 31% of scientists felt that research university systems value research productivity, as indexed by grants and published papers, over everything else, including outreach. With this prioritization structure in place outreach may be perceived as unrelated to a scientist’s academic pursuits. Additionally, the lack of funding and opportunity can make outreach labor intensive. Although social media outreach is perceived to be easy and low cost in terms of money and time, success of such endeavors may require considerable investment. Using Deep-Sea News, which garnered 2.5 million hits in 2013 as an example, the lead author spent approximately 588 hours in 2013 on social media outreach translating into 14.7 40-hour workweeks.

The “Sagan Effect” - professional stigma associated with public engagement – is perceived to be widespread ^29^. Even those who support active engagement frequently assume that a scientist’s research quality is inversely proportional to the amount of outreach work he or she does. Interestingly, Sagan’s own research productivity in terms of published scientific articles remained constant, ~10 annually, throughout his career and his total scientific body of work is comparable to other eminent scientists ^46^. However, recent changes by funding agencies, professional agencies, and scientist’s views toward public outreach may help to diminish the “Sagan Effect”. Moreover, the number of scientific citations is greater among French scientists who engage in greater public dissemination activities ^28^. In terms of social media, there also appears to be an overall positive correlation between the number of citations and number of Twitter followers among scientists ^47^.

However, one way that the social media appears not to impact a scientific career is a direct link of social media mentions and citations on a scientific article. In an analysis of 1.4 million documents in PubMed and Web of Science published from 2010 to 2012, Haustein *et al.* ^48^ found no correlation between a paper or a journals citation count and Twitter mentions. As argued by the authors of the study, this suggests that Twitter mentions do not reflect traditional research impact. Rather, social media mentions may capture a previously unquantified impact of a scientist’s career ^49^.

Although, a direct link between SOSM and benefit to scientists career may not been supported, this does not rule out indirect effects such as seeing practical applications of research, increasing overall skills in communication, or gaining expertise in additional areas of science, i.e. becoming more well rounded. Unfortunately, indirect effects in any system are difficult to quantify and assess but ripe for further research.

## Can and should SOSM be integrated into a scientist’s career?

We argue that actions and rhetoric intended to support or encourage SOSM must focus on the return on investment. For the equation to work out in favor of wide-spread and career-long engagement, we propose the following three elements are necessary:

**1. It must be valued:** The reward for SOSM is often seen as a direct impact on the metrics that are used in gauging scientific success, e.g. gaining tenure or earning promotion. As outlined above, the sparse data available suggest these impacts on publications, citations, and grants may be minimal and indirect. A conversation about SOSM, and outreach in general, is needed that focuses on the metrics of scientific success used by both the community and institutions and developing new ones. Recently, the University of Wisconsin-Madison Division of Biological Sciences, WI, United States, has enacted new tenure guidelines that place greater reward and priority for outreach.

*“Excellence can be documented in [these] clearly defined areas, and/or through synergistic combinations of research, teaching, and outreach or service… A key component for excellence in outreach is the dissemination of information derived from scholarly inquiry for the benefit of society. Successful outreach will involve innovative practices, program developments, impacts and applications that have made continuing and substantial contributions at the local, regional, national or international level. It may also lead to transformative practices derived from clinical programs or community engagement for the benefit of society. A demonstrated capability to develop and sustain an independent, cohesive, and impactful outreach program is essential. Dossiers must include documentation of the outcomes of outreach and its impacts and, in addition, include evaluations by recognized outreach specialists in the candidate’s field outside UW-Madison”*.

Tenure guidelines for the University of Illinois-Urbana Champaign, IL, United States, also place an increasing emphasis on “valuable public engagement” though excellence in outreach is not enough to warrant tenure.

We must expand the conversation beyond “traditional” tenure-track positions. Scientists working in the private and government sectors often do not enjoy the same freedom to actively engage the public. Scientists employed by the government are often not allowed, or highly constrained, in how they engage the public especially in the new online frontier that is social media. In the most severe case, the Environmental Protection Agency has sought to increase restriction even on those independent scientists who advise the agency, a movement opposed by journalists and scientists alike ^50^. At the United States Geological Service, the use of social media “to regularly talk about your area of expertise” must be “approved by Office of Communications and Publishing and your supervisor”.

One area of information needed is whether institutions and scholars treat SOSM activities as actual academic and beneficial pursuits. In terms of social media as inreach, many researchers within academia may not treat blogs as serious academic products because they are not peer reviewed ^51^. Geoffrey North in *Current Biology* stated, “But there is also, I think, a danger here, which lies in the very speed of response, and the way that blogs are essentially “vanity publications” which lack the constraints of more conventional publishing — they are not reviewed, and do not even have to pass the critical eye of any editor” ^52^.

Conversely there may be valid reasons to keep, social media, particularly inreach blogging, free from formal academic systems. Blogging may allow a circumvention of hierarchy and power dynamics of academia ^53^ and provide a space “distinct from the parent culture of institutions” ^54^. In terms of both inreach and outreach, anonymity or pseudo-anonymity have often provided underrepresented groups and junior scientists the sense of security needed to discuss issues in science openly. However, while bringing needed voices to the conversation, these scientists do not get to reap any formal rewards within the halls of academia. Clearly SOSM and more generally the usage of social media require reassessment of what defines academia, scholars, scholarship, and institutions. Part of this will also have to address power dynamics in academia that prevent underrepresented groups and junior faculty from pursuing innovative online models for outreach.

**2. It must be measured:** We have watched with great interest the recent emergence of Altmetrics as a solution to accurately representing the totality of a scientist’s scholarly impact in a web-native world ^49^. Quantification of total downloads and social media mentions for individual scientific papers have provided an exciting advancement in measuring influence. However, these metrics do not yet capture the total social media and web presence for a researcher, e.g. blog posts or Tweets, meaning there is not yet digital equivalency for the H-index. Furthermore, as with traditional metrics, current tools cannot discriminate between positive and negative mentions (citations), and with the dynamics of social media sharing at play, great care must be taken not to conflate ‘activity’ metrics with proof of influence or approval. The need to consider both quality and quantity was ostensibly the intent of the satirical attempt ^47^ to calculate the so-called Twitter Kardashian Index by plotting individual researchers’ numbers of followers against the number of citations of their scientific output and thereby identify those who are more “famous” than their publication record merits. We find the development of a K-Index, whether in satire or not, to be counterproductive to meaningful understanding of the role of social media and fraught with methodological problems. Indeed, instead of criticizing scientists with large number of Twitter followers, we should be studying them as exemplars for that dimension of social media success.

**3. It must be manageable:** The most identified issue by scientists preventing them from engaging in SOSM, and outreach in general, is a lack of time. This issue remains relatively unaddressed. If the goal is ultimately to have more scientists engaged more fully in outreach through social media there is a need to target the conflict of the clock. One possible mechanism for this is a reprioritization and integration of research and outreach.

One way forward may be finding opportunities where effort and products can serve more than one purpose. Several existing examples demonstrate that a research program and SOSM can be integrated. For example, blog posts on scientific literature already being read for journal clubs, classes, grants, and publications represent both a way to more actively engage the literature scientifically while producing online content for public consumption. Crowd funding of science both directly engages the public while also serving as a mechanism to generate smaller funds for research ^55, 56^. Online content can also be generated as a product of classroom activities. For example, the “Sizing Ocean Giants” project ( http://www.storyofsize.com/sizing-ocean-giants/), an undergraduate directed research course, focused on the body sizes of marine organisms and included a heavy social media component. Undergraduates were directed to use social media to engage the public about their specific animals, leveraging online platforms as a pedagogical tool to increase their understanding of their organisms and the scientific process. As with traditional courses, writing assignments on a variety of scientific topics were included, but these were simply migrated online as blog posts. Twitter assignments included #sizeme where the public was encouraged to Tweet height or weight and students in the course responded with how the measurements compared to a species of marine megafauna effectively illustrating scale. Ultimately, the research by students benefited from online interactions with other scientists and lead to a peer-reviewed publication.

Many programs also exist within and outside of universities for scientists to engage in science communication and SOSM. These require less effort on the part of individual academics because infrastructure, and often training, are already in place. For example, Experiment.com provides a mechanism for scientists to launch crowd-funding campaigns. Setting out to create a new blog may not be as effective as providing a blog post to an established blog or joining a group blog.

## Conclusion

We clearly need a new way to consider, conduct, and evaluate science outreach, online and off. This new model must be informed by theory and grounded in data analysis, as opposed to predicated on anecdote and assumption. We have shown here that many popular propositions about the attitudes, behaviors, and actions of scientists engaged in SOSM are incorrect. These conceptual errors may slow or impede progress by diverting efforts into solutions for the wrong problems. We propose efforts should concentrate on making SOSM a valued and worthwhile endeavor within and outside academia, an actual quantification of the value of SOSM by individuals, and creating programs that make SOSM time manageable for scientists.
