# Supplementary material for: A critical evaluation of science outreach via social media: its role and impact on scientists
Source: F1000Res. 2015 May 18;3:300. Originally published 2014 Dec 9. [Version 2] doi: 10.12688/f1000research.5918.2 (PMC4376169; doi:10.12688/f1000research.5918.2)
Supplement: Peer review history

# Peer review: Referee response for version 1

**DOI:** 10.5256/f1000research.6322.r7921

**Published:** 2015-03-26

**Authors:** Sharon L. Dunwoody

**Affiliations:** 1 School of Journalism and Mass Communication, University of Wisconsin‐Madison, Madison, WI, USA

Readers of this opinion piece will appreciate its ability to locate and reflect on much of the recent literature dealing with the extent and perceived impact of scientists' outreach behaviors. That literature gives social media only a glancing blow, so the authors are correct to note that we know little about scientists' use of social media and about perceived and actual outcomes of such use. We do know that social media use is growing among scientists, and mainstream empirical studies - which typically generate snapshots in time that won't become available in published form for months (or years) - will have a hard time staying on the front edge of such changes. McClain and Neeley's analysis leads me to suggest that scholars pay particular attention to the impacts of social media outreach on scientists' careers. While we know that rendering one's research publicly visible via such traditional means as mass media stories has a salutary impact on the number of citations to that work in the peer-reviewed literature, we are just beginning to track the impact of scientists' "unmediated" signaling via tweets, blogs posts, etc. Some recent work found that TED talks did not appear to make much of a difference.  But a very recent article in *Journalism & Mass Communication Quarterly* ( Liang *et al*., 2014) did find that, among a sample of nanoscientists, tweeting was associated with more frequent interactions with reporters and other non-scientists and that those interactions were then linked to enhanced scientific impact. I would implore the authors to "step away" from the acronyms. SOSM (science outreach via social media) deserves a quick burial. As is the case with so many acronyms, it obfuscates rather than clarifies.

I have read this submission. I believe that I have an appropriate level of expertise to confirm that it is of an acceptable scientific standard.

# Peer review: Referee response for version 1

**DOI:** 10.5256/f1000research.6322.r7923

**Published:** 2015-03-20

**Authors:** Kathryn B. H. Clancy

**Affiliations:** 1 Department of Anthropology, University of Illinois, Urbana, IL, USA

McClain and Neeley make an important contribution to the science communication literature with their opinion article “A critical evaluation of science outreach via social media: its role and impact on scientists.” The authors do a great job dismantling many of the misconceptions held by those who naysay science communication. I also loved their manifesto at the end of the three necessary elements to support social media for science outreach (particularly the last element, which is very consistent with some initiatives on work allocation I am currently attempting at my own institution!). I have only a few comments about ways in which these authors could strengthen their manuscript:

- The introduction could more clearly state the authors’ argument. As experts in this field, the authors could really frame their argument for the reader by offering it from the beginning. Right now the introduction does not make clear what side of the issue for which the authors advocate.
- Related to this: the headings, instead of asking the question, could just make your point. So for instance, the first could read “Science outreach is not a “social media nightmare.”” For the browsing reader, making your point and THEN iterating it can be powerful.
- I love how strongly cited this article is. I would love to see a little more of the extended explanation of the empirical articles to which the authors cite in their first section – they do a great job of this in the other sections.

Minor issues:

- The acronym “SOSM” appears to be out of order, since it stands for Social Media for Science Outreach (SMSO).
- Personally I’m not crazy about using “impact” when the intended meaning is “effect,” as is used in the title, but I leave that very minor decision up to the authors.
- When there is an extended quote, it really should be turned into a blockquote so that it’s clear to the reader the authors are using the words of another to strengthen their argument (examples: first and second paragraphs in right column of page 3). Though I also felt these extended quotes might be better paraphrased by the authorsso as to avoid patchwriting.
- The use of the term “academic inclusion” (page 4, second full paragraph, right column) is confusing. Inclusion in academic circles is usually a term to discuss diversity initiatives.

Thanks for the opportunity to review this interesting piece.

I have read this submission. I believe that I have an appropriate level of expertise to confirm that it is of an acceptable scientific standard.

# Peer review: Referee response for version 1

**DOI:** 10.5256/f1000research.6322.r7573

**Published:** 2015-02-18

**Authors:** Paige Brown Jarreau

**Affiliations:** 1 Manship School of Mass Communications, Louisiana State University, Baton Rouge, LA, USA

This article provides a much needed point of departure for future research regarding scientists' engagement in social media and public engagement. This article raises great questions that will need answering through future empirical evidence.

Previous studies have often queried scientists' engagement in social media and public outreach on a broad level. However, "scientists" is an extremely broad grouping. There has been some work looking at scientists' engagement in social media and public outreach by discipline and demographic variables including age and gender, but we need to go further than this. Why do some scientists make the time to engage in outreach or social media, such as tweeting and blogging, while others do not? Logistical constraints are probably involved, but are likely not the major constraint, as many extremely busy scientists find time or make time to engage in outreach via social media. How does the culture at specific organizations/institutions affect this engagement, or scientists' decisions of whether it is "worth it"? And how can social scientists uncover these nuances of behavior with regards to scientists' use of social media for outreach? I think it would be valuable it the authors addressed these questions, and addressed how future research should focus on nuances vs. overly broad descriptions of scientists' use of social media for outreach.

In the section on how social media outreach will benefit scientists' careers, the authors focus on tangible and professional benefits that are compatible with traditional measures of scientific/academic performance (such as citations and tenure). This leaves out more intangible benefits that scientists may experience from engaging in social media outreach, such as being able to see the practical applications of their research, or becoming more well-rounded in terms of their scientific expertise outside of their narrow fields of research. The authors should address how less traditional "benefits" of social media outreach can be measured and communicated to scientists in various fields. A look at the degree of collaboration that occurs for researchers who don't engage in social media vs. those who do would also be interesting.

A few other notes:

1. "the best predictor of outreach by scientists appears to be linked to their perceptions of outreach" - why isn't this explained in the context of the theory of planned behavior, or the value-belief-norm model for explaining scientists' behavior toward social media engagement / outreach?
2. "Moreover blogs written by scientists for scientists are becoming common and important places for the exchange of ideas" - I would caution against broad statements about the impacts of blogs, or what kind of blog genres are growing. In fact, there is some evidence, including from my own ongoing research (although more research is needed), that blogs geared toward outreach and explaining science to broader audience are outnumbering / growing past those blogs that are written by scientists for other scientists (these types of blogs are generally in the minority). Just as in everything else, we need more data about science blogging approaches and the impacts of those approaches. For example, which different social networks (blogs? Twitter? ResearchGate and similar science-focused social networks) are being used by researchers to talk to other researchers, and which are being used in a more outreach type of capability, and why?

I think the authors could round-out their discussion of needed research in this and related areas through a modest revision of their original article. I also encourage the authors to discuss how theories of mass communication, psychology and behavior could fit into the areas of research they call for.

I have read this submission. I believe that I have an appropriate level of expertise to confirm that it is of an acceptable scientific standard, however I have significant reservations, as outlined above.
